# Prediction of cardiovascular disease risk using biomarkers in blood

**DOI:** 10.6026/973206300211547

**Published:** 2025-06-30

**Authors:** Sruthi Vempatapu, Daleena Prathiba Madda, Akhil N., Kongara Sree Nischal Chowdary

**Affiliations:** 1 Department of Emergency Medicine, Yashoda hospitals, Hyderabad, Telangana, India; 2 Department of General Medicine, New Vara Hospital, Hyderabad, Telangana, India; 3 Department of General Medicine, Kakatiya Medical College, Warangal, Telangana, India; 4 Department of General Medicine, Dr.Pinnamaneni Siddhartha Institute of Medical Sciences & Research Foundation, Chinna Avutapalli, Vijayawada, Andhra Pradesh, India

**Keywords:** Cardiovascular disease, CRP, LDL cholesterol, IL-6, Biomarkers, Risk prediction

## Abstract

Prediction of cardiovascular disease risk using biomarkers in blood is of interest. Hence, evaluation of blood biomarkers as predictors of cardiovascular disease (CVD) among 300 participants is completed. C-reactive protein (CRP), Troponin I, LDL, HDL, and IL-6 levels were measured, showing significant differences between CVD and non-CVD groups. CRP and Troponin I demonstrated the highest predictive accuracy with AUCs of 0.89 and 0.85. A combined model using CRP, Troponin I, and LDL showed 88% sensitivity and 84% specificity. These biomarkers may aid early CVD detection and risk stratification in clinical practice.

## Background:

Despite global advances in cardiovascular healthcare, cardiovascular diseases (CVDs) remain the leading cause of mortality, contributing to over 17.9 million deaths annually, according to WHO estimates-notably surpassing the combined burden of all communicable diseases. However, this high burden is not uniformly distributed across all CVDs. For example, atrial fibrillation (AF), a common arrhythmic manifestation of CVD, significantly increases stroke risk and overall mortality [1]. Early and precise identification of individuals at high risk for CVD is paramount to improving clinical outcomes and targeting preventative interventions more effectively. Recent studies have increasingly emphasized the clinical utility of cardiac and inflammatory biomarkers for stratifying CVD risk. High-sensitivity Troponin I (hs-TnI), a marker of myocardial injury, has been shown to independently predict stroke, myocardial infarction, and cardiovascular death in patients with stable and acute coronary syndromes, even when no overt symptoms are present [2, 3]. Elevated troponin levels have been independently associated with poor outcomes in AF populations as well [1, 4]. In parallel, C-reactive protein (CRP), a hepatic acute-phase reactant, provides robust prognostic information about systemic inflammation and vascular events, including myocardial infarction and ischemic stroke [5, 6].

A growing body of evidence supports the role of CRP in refining CVD risk scores and monitoring treatment efficacy [7]. Further, interleukin-6 (IL-6), a pleiotropic pro-inflammatory cytokine, has been linked with endothelial dysfunction, plaque instability, and poor prognosis in both AF and coronary artery disease patients [8, 9]. Elevated IL-6 levels have been independently associated with thromboembolic complications and cardiovascular mortality in large cohorts [10, 11]. While IL-6 plays a regulatory role in immune and inflammatory pathways, its clinical utility in routine CVD risk stratification remains under-explored and warrants further validation. In the lipid spectrum, LDL-C contributes directly to atherosclerotic plaque formation, while HDL-C confers vascular protection via reverse cholesterol transport and anti-inflammatory effects [13, 14]. Dyslipidemia, especially a high LDL-C to HDL-C ratio, remains a strong predictor of ischemic events, particularly when combined with elevated inflammatory markers [15]. Although several studies have individually evaluated these biomarkers, this study uniquely integrates CRP, hs-Troponin I, IL-6, LDL-C, and HDL-C into a combined predictive model, applying Receiver Operating Characteristic (ROC) curve analysis and multivariate regression modeling to determine optimal biomarker combinations for clinical application. This approach provides a precision-based advancement by identifying high-sensitivity, high-specificity biomarker panels rather than relying on isolated indicators. Moreover, the study fills a translational gap by assessing these markers in a general population aged 40-70, thus enhancing their applicability in routine clinical risk assessment protocols. Therefore, it is of interest to evaluate the diagnostic accuracy of CRP and Troponin I, and to investigate the complementary role of IL-6, LDL-C, and HDL-C in predicting cardiovascular disease risk. By employing integrated biomarker analysis via ROC curves and multivariate models, this research seeks to establish a clinically applicable biomarker panel that enhances early detection and risk stratification in cardiovascular disease.

## Materials and Methods:

Three hundred participants participated in this study which included 150 patients with medically confirmed CVD and 150 individuals who did not suffer from cardiovascular diseases. Ethical approval from the Institutional Review Board (IRB) along with participant consent guided the recruitment process of participants from Yashoda Hospital, Hyderabad.

## Inclusion and exclusion criteria:

The research included participants between the ages of 40-70 who were divided into two groups based on their status: patients were selected from patients diagnosed with cardiovascular disease following clinical evaluation and laboratory or imaging diagnosis. The control subjects were selected from people without previous medical conditions of CVD along with diabetes and chronic inflammatory diseases. The research excluded subjects with autoimmune diseases and those with chronic infections, malignancies or participants who received anti-inflammatory or immunosuppressive therapy.

## Sample collection and biomarker analysis:

All blood testing (5 mL) began after participants spent the night without eating. The medical staff drew blood through venepuncture from each study participant. Serum obtained by centrifugation at 3000 rpm during 10 minutes storage went into -80°C freezers until additional experiments started. The research tested biomarkers composed of C-reactive protein (CRP), Troponin I, low-density lipoprotein cholesterol (LDL-C), high-density lipoprotein cholesterol (HDL-C) and Interleukin-6 (IL-6). Analysis of CRP levels occurred through high-sensitivity enzyme-linked immunosorbent assay (hs-ELISA) methods and Troponin I measurements used chemiluminescent immunoassay (CLIA) systems. The enzymatic colorimetric procedures measured quantitative concentrations of LDL-C in combination with HDL-C. Commercial ELISA kits allowed the assessment of IL-6 levels according to manufacturer specifications.

## Statistical analysis:

The statistical analysis was conducted through SPSS 26. The researchers employed descriptive statistical methods to present essential characteristics about the study participants. The comparison of biomarkers between CVD patients and control subjects relied on independent t-tests and Mann-Whitney U tests based on data distribution normality. Predictive performance of each biomarker was determined through ROC curve analysis that applied AUC statistics as diagnostic indicators. Regression modeling techniques with multiple variables helped evaluate predictive biomarker patterns. The diagnostic properties of biomarkers were evaluated by calculating sensitivity together with specificity and positive predictive value and negative predictive value. The analysis determined statistical significance when p-value fell below 0.05.

## Results:

The study included 300 participants, comprising 150 patients with confirmed cardiovascular disease (CVD) and 150 healthy controls. The mean age of the participants was 56.2 ± 8.4 years, with 60% males and 40% females distributed evenly across the groups.

## Biomarker levels in CVD and control groups:

The levels of C-reactive protein (CRP), Troponin I, low-density lipoprotein cholesterol (LDL-C), high-density lipoprotein cholesterol (HDL-C), and Interleukin-6 (IL-6) were assessed in both groups. As shown in Table 1 (see PDF), the CVD group exhibited significantly higher levels of CRP, Troponin I, LDL-C, and IL-6 compared to the control group (p < 0.05). Conversely, HDL-C levels were significantly lower in the CVD group compared to the controls (p < 0.05). The significant differences observed in the levels of CRP, Troponin I, LDL-C, HDL-C, and IL-6 suggest their potential utility in distinguishing CVD patients from healthy individuals (Table 1 - see PDF).

## Predictive accuracy of biomarkers:

Receiver Operating Characteristic (ROC) curve analysis was performed to evaluate the diagnostic accuracy of individual biomarkers. The area under the curve (AUC) values for each biomarker is presented in Table 2 (see PDF). CRP and Troponin I demonstrated the highest predictive accuracy for CVD, with AUC values of 0.89 and 0.85, respectively. The findings from the ROC analysis indicate that CRP and Troponin I are the most reliable biomarkers for predicting CVD risk (Table 2 - see PDF).

## Multivariate regression analysis:

A multivariate regression model combining CRP, Troponin I, and LDL-C was developed to enhance predictive accuracy. The model achieved a sensitivity of 88% and a specificity of 84%, indicating its potential clinical utility for risk prediction.

## Discussion:

Blood biomarkers show significance as CVD risk indicators due to their predictive power via C-reactive protein (CRP) and Troponin I assessment. Results from this study demonstrate parallel findings to previous research which shows CRP, Troponin I, LDL-C, and IL-6 elevation together with decreased levels of HDL-C occurs in subjects with CVD [1, 2-3]. Apparently healthy individuals can depend on CRP since this inflammation-mediated acute-phase protein serves as an established tool for predicting future cardiovascular events [4]. Research evidence indicates the predictive effectiveness of the area under the curve value at 0.89 which shows the clinical value in assessing CVD risk [5]. CRP plays a direct role in the development of endothelial dysfunction and atherosclerosis which supports its strong link to CVD [6]. Doctors widely recognize Troponin I as a highly specific marker because it diagnoses myocardial injury accurately in acute coronary syndromes according to medical research [7]. Research from the current study confirmed past studies which showed that measurements help predict dangerous cardiac occurrences [8]. The CVD group showed raised Troponin I levels together with an AUC value of 0.85 which demonstrates its powerful predictive capabilities for CVD [9]. The development of atherosclerosis depends heavily upon dyslipidemia mechanisms because elevated LDL-C along with lowered HDL-C levels is extensively documented in medical literature [10]. Oplasmic lipids under LDL-C control participate in plaque growth which blocks arteries but elevated HDL-C helps remove cholesterol and protect blood vessels from oxidative stress [11, 12]. Previous studies have validated our research findings of elevated LDL-C and reduced HDL-C in CVD patients while showing their significance for CVD risk determination [13]. Research links the pro-inflammatory cytokine IL-6 to CVD development because it promotes endothelial dysfunction as well as plaque instability while contributing to sustained inflammation [14]. Research findings indicate that elevated IL-6 levels in CVD patients confirm its potential value for predictive assessment [15]. The study used multivariate regression to confirm the predictive model based on CRP along with Troponin I and LDL-C has sensitivity at 88% and specificity at 84%. Better prediction rates for CVD risk can be achieved through using multiple biomarkers instead of using a single marker according to these study findings [1]. The use of multiple biomarkers in diagnosis shows alignment with the rising precision medicine trend since this method improves diagnostic precision [7]. The results of this research face several restricting factors. The research design types as well as the studied population representation maintain significant constraints on establishing cause-effect relationships and achieving full demographic inclusivity. This study failed to assess two emerging biomarkers namely lipoprotein-associated phospholipase A2 (Lp-PLA2) along with N-terminal pro-B-type natriuretic peptide (NT-proBNP) even though they may have provided additional predictive value [8, 9]. Longitudinal research involving extensive and diverse test groups must be conducted to confirm current results and assess practical values of integrating multiple biomarker analysis models [2].

## Conclusion:

Blood biomarkers particularly CRP and Troponin I demonstrate strong potential as reliable measures of cardiovascular disease risk status. Routine clinical procedures that include these biomarkers will help professionals detect risk early so they can take appropriate action for people at high risk. Additional research needs to be conducted to enhance predictive models as well as make them available to multiple population groups.
